# Biological Synthesis of Bioactive Gold Nanoparticles from *Inonotus obliquus* for Dual Chemo-Photothermal Effects against Human Brain Cancer Cells

**DOI:** 10.3390/ijms23042292

**Published:** 2022-02-18

**Authors:** Ibrohimjon Shukurov, Mohamed Sheikh Mohamed, Toru Mizuki, Vivekanandan Palaninathan, Tomofumi Ukai, Tatsuro Hanajiri, Toru Maekawa

**Affiliations:** 1 Graduate School of Interdisciplinary New Science, Toyo University, Kawagoe 350-8585, Japan; ibrohimshukurov666@gmail.com (I.S.); mizuki@toyo.jp (T.M.); vivekanandan@toyo.jp (V.P.); tmfmukai@toyo.jp (T.U.); hanajiri@toyo.jp (T.H.); maekawa@toyo.jp (T.M.); 2 Bio-Nano Electronics Research Centre, Toyo University, Kawagoe 350-8585, Japan

**Keywords:** gold nanoparticles, *Inonotus obliquus*, biological synthesis, brain cancer, anti-cancer, chemotherapy, photothermal therapy, biofunctionalization, bioactivity

## Abstract

The possibility for an ecologically friendly and simple production of gold nanoparticles (AuNPs) with Chaga mushroom (*Inonotus obliquus*) (Ch-AuNPs) is presented in this study. Chaga extract’s reducing potential was evaluated at varied concentrations and temperatures. The nanoparticles synthesized were all under 20 nm in size, as measured by TEM, which is a commendable result for a spontaneous synthesis method utilizing a biological source. The Ch-AuNPs showed anti-cancer chemotherapeutic effects on human brain cancer cells which is attributed to the biofunctionalization of the AuNPs with Chaga bioactive components during the synthesis process. Further, the photothermal ablation capability of the as-prepared gold nanoparticles on human brain cancer cells was investigated. It was found that the NIR-laser induced thermal ablation of cancer cells was effective in eliminating over 80% of the cells. This research projects the Ch-AuNPs as promising, dual modal (chemo-photothermal) therapeutic candidates for anti-cancer applications.

## 1. Introduction

Chaga or birch fungus is a birch mushroom, which is a waste product of the wood-destroying polypore fungus *Inonotus obliquus*, that parasitizes on the trunks of living trees (birch, less often mountain ash, alder). Extracts based on it are widely employed in the therapy and preventive measures against a plethora of ailments, such as gastrointestinal, oncological and a number of other diseases [1,2,3,4]. In addition, Chaga and its extracts have interesting biological activity, exhibiting antioxidant, gene-protective, immunomodulatory, antiviral, antitoxic and radioprotective properties [5,6]. The mushroom has found prominent place in folk medicine in the Baltic countries, Russia, Ukraine, Poland and Belarus since the 16th century for the treatments of cancer, cerebrovascular diseases, diabetes and tuberculosis [7,8,9,10,11]. In Eastern European countries and the North American regions it has been known to be used to treat heart ailments as well. Reports even exist on the therapeutic effects of *I. obliquus* in the prevention and cure of AIDS, positive regulation of blood lipids and blood pressure, anti-aging effects and its ability to boost the body’s immune system [12,13,14]. Apart from the anti-oxidant, anti-inflammatory, hypoglycemic, anti-mutagenic and anti-nociceptive properties of *I. obliquus,* its antitumor activity has also been reported [7,15,16,17]. The main reason for the multifarious medicinal potentials of this mushroom is due to the fact that the extracts of *I. obliquus* are rich in bioactive components, such as inotodiol, betulinic acid, oxygenated triterpenes, superoxide dismutase (SOD), tannin compounds, alkaloid, lignin, folic acid derivatives, polyphenols, aromatic substances, peptides, polysaccharides, polyphenols, triterpenoids and steroids [18,19,20,21,22,23,24]. 

Compared with those of metal atoms or bulk metals, nanomaterials have gained significant interest because of their peculiar optical, chemical, photoelectrochemical, and electrical properties [25,26,27,28]. In products ranging from cosmetics to pharmaceuticals, noble metal nanoparticles (NPs), such as gold, silver, platinum and palladium, have been commonly used. Of these, much of the attention has been devoted to gold nanoparticles (AuNPs) due to their intriguing features as size, shape, high biocompatibility and surface plasmon resonance effect due to which, AuNPs have been widely utilized in the field of theranostics, which encompasses the ability of a material to perform both diagnostic abilities and therapeutics [29,30,31]. AuNPs also play a prominent role in the alternative therapy regimen, which include therapeutic options other than the conventional clinical practice (surgery, chemotherapy, radiotherapy). One such alternative therapy is photothermal therapy (PTT) which is triggered by an external stimulus, such as light. PTT generates localized rapid heating effect, which could be utilized to destroy a cancer mass. This particular property can be utilized in combination with the conventional therapeutic modules or as an independent treatment strategy [32,33,34,35,36,37]. 

Tremendous research has been conducted on the synthesis of AuNPs, both chemical and biological [38,39,40,41,42], to explore the various options for determining the best mode for producing size and shape tuned, biocompatible nanoparticles. Herein, we study the effects of an aqueous extract of Chaga mushroom on its ability to act as a reducing and capping agent for the AuNPs. The as-synthesized AuNPs (Ch-AuNPs) were characterized for their bioactivity and PTT properties against different brain cancer cells.

## 2. Results and Discussion

### 2.1. UV-Vis Characterization of Ch-AuNPs

**Figure 1:**
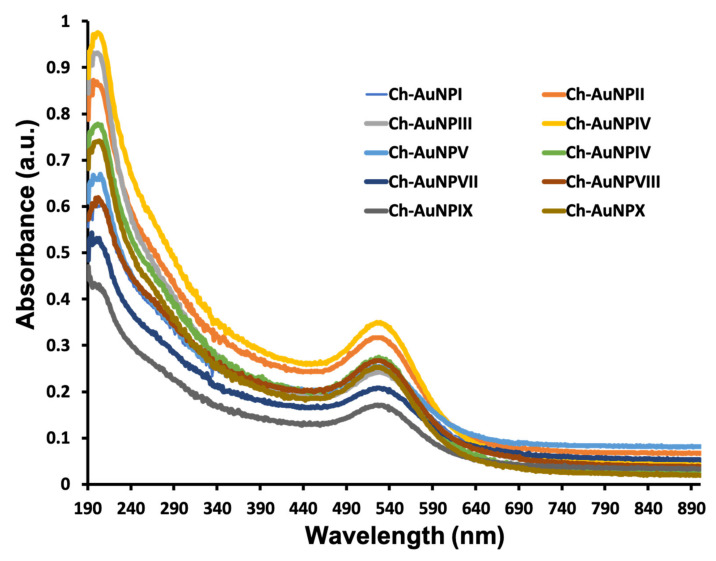
UV-Vis spectra of the as-synthesized Ch-AuNPs. The typical absorption region for AuNPs (500–600 nm) can be clearly witnessed with peak maxima centered about 520 nm. The spectra were almost similar for all the reaction conditions’ obtained Ch-AuNPs.

The formation of gold nanoparticles by HAuCl_4_ reduction with Chaga extract was indicated by associated change in the color of the reaction mixture. After 24 h, the color of the reaction solution transformed from dark brown to reddish brown. As a result of extensive screening with different concentrations of auric chloride and Chaga extract, synthesis was successful in both concentration ratios and all temperature ranges. The UV-Vis spectra of the suspension revealed a typical absorption (500–600 nm) with peak maxima centered about 520 nm (Figure 1), which is a signature of the AuNP’s surface plasmon resonance (SPR) band.

### 2.2. Morphological Characterization of Ch-AuNPs

**Figure 2:**
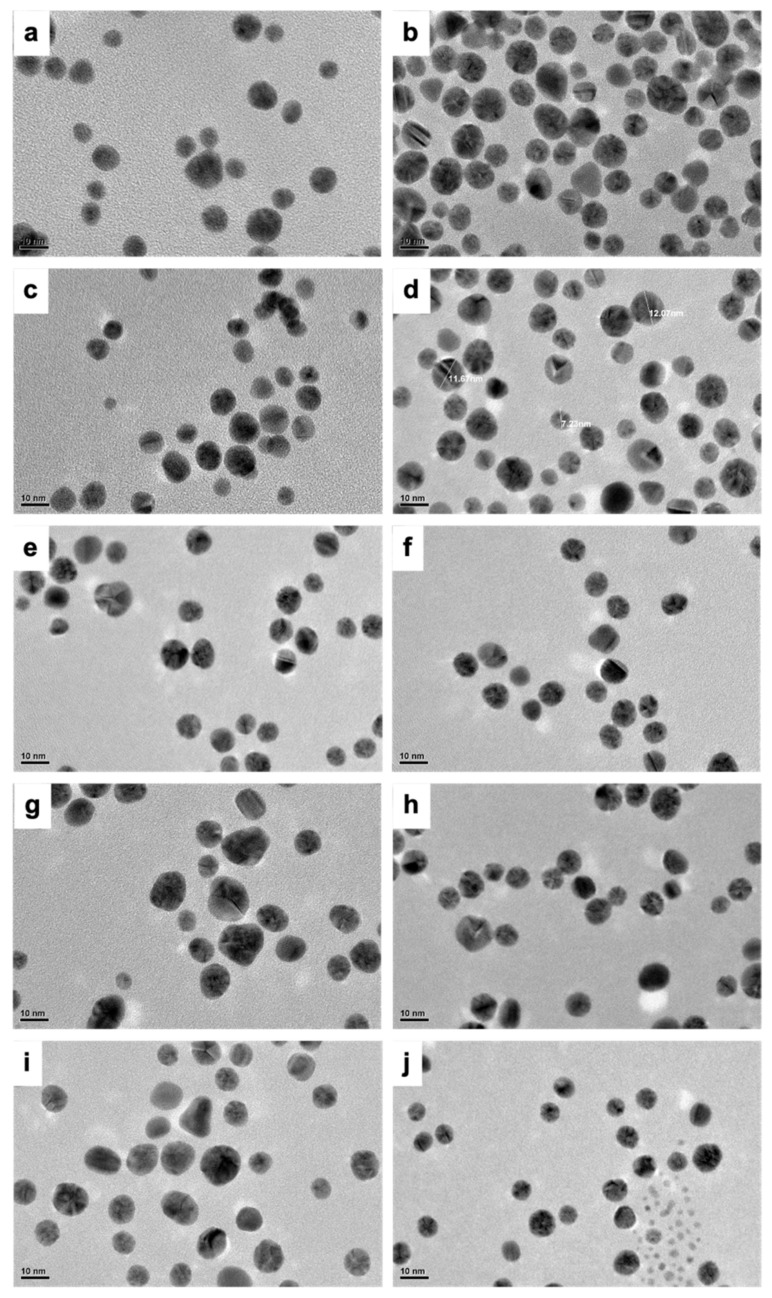
TEM micrographs of the Ch-AuNPs synthesized under various parameters. (**a**) Ch-AuNPI, (**b**) Ch-AuNPII, (**c**) Ch-AuNPIII, (**d**) Ch-AuNPIV, (**e**) Ch-AuNPV, (**f**) Ch-AuNPVI, (**g**) Ch-AuNPVII, (**h**) Ch-AuNPVIII, (**i**) Ch-AuNPIX, (**j**) Ch-AuNPX. It could be observed that though there is a slight diversity in shape and size of the NPs, overall, the NPs are uniform in their morphological features. (scale bar = 10 nm).

In most of the samples, TEM results demonstrated the presence of differently shaped nanoparticles which included triangles, spherical and ellipsoidal particles (Figure 2). However, it was found that most of the nanoparticles synthesized were spherical in nature. Interestingly, nearly all the nanoparticles were less than 20 nm in size (Figure S1). 

This is a truly intriguing outcome, as the size and shapes of the nanoparticles are widely varied in most processes requiring the use of natural sources for nanoparticle synthesis. It is therefore a commendable accomplishment to obtain nearly uniform shape and sized (~20 nm) nanoparticles with a biological synthesis method. Numerous reports exist of utilizing natural resources for the production of AuNPs. Results pertaining to the size and morphological features of the synthesized nanomaterials vary depending mainly on the type of biological source chosen for the synthesis, followed by the working concentration utilized and related synthesis parameters, such as temperature, pH, etc. [43,44,45,46,47,48]. Though the latter parameters play an important part, it is a unanimous conclusion that the choice of the reducing biological source is crucial, with great emphasis on the biomolecular content which has a determining impact on the morphological characteristics of the NPs. In our case, as explained, Chaga is extremely high in phytomolecules, thus providing a rich source for AuNPs synthesis and possible capping as well. It is therefore perceived that the highly bioactive molecules of Chaga played an important role in deciding the size and shape related features of the Ch-AuNPs. 

Table 1 provides, in addition to that of the extract, the hydrodynamic size and zeta potential of the Ch-AuNPs as measured by dynamic light scattering (DLS). Nearly all of the nanoparticles were under 50 nm. It is well established that the zeta size is a measure of the particles’ hydrodynamic size that is marginally greater than the real TEM observation. Therefore, the size measurements were consistent with the TEM observations. Interestingly, the polydispersity index (PI) of the synthesized NPs was also in the acceptable range. The PI provides the degree of uniformity/heterogeneity of a test sample solution determined by the size of the particles in the solution. According to international standards organizations (ISOs—ISO 22,412:2017 and ISO 22,412:2017), a larger PI value (>0.7) denotes a more polydisperse particle population which can be a result of varied size, agglomeration or aggregation of the sample, whereas a PI value of <0.05 represents monodispersity and uniformity [49,50]. All the samples Ch-AuNPI-X displayed a PI of <0.6. Additionally, the zeta potential measurements showed values for all nanoparticles of around −25 mV implying a good and stable colloidal suspension devoid of aggregates. A good zeta potential value indicates good colloidal stability of the samples [51].

Therefore, based on the TEM and zetapotential observations it can be confidently stated that the Ch-AuNPs synthesized using Chaga extract do not form aggregates, and rather have a high degree of colloidal stability. It could be seen that the potential value of the extract itself was much greater (−40) which is due to the numerous biomolecules that make up the extract.

### 2.3. Elemental Characteristics of Ch-Extract and Ch-AuNPs

In order to classify the organic-inorganic interfaces, XPS studies of Chaga extract and Ch-AuNPs were carried out and the findings obtained are shown in Figure 3 and Figures S4–S11. The spectrum of the survey suggests the existence of Au, C, O, N and K.

**Figure 3:**
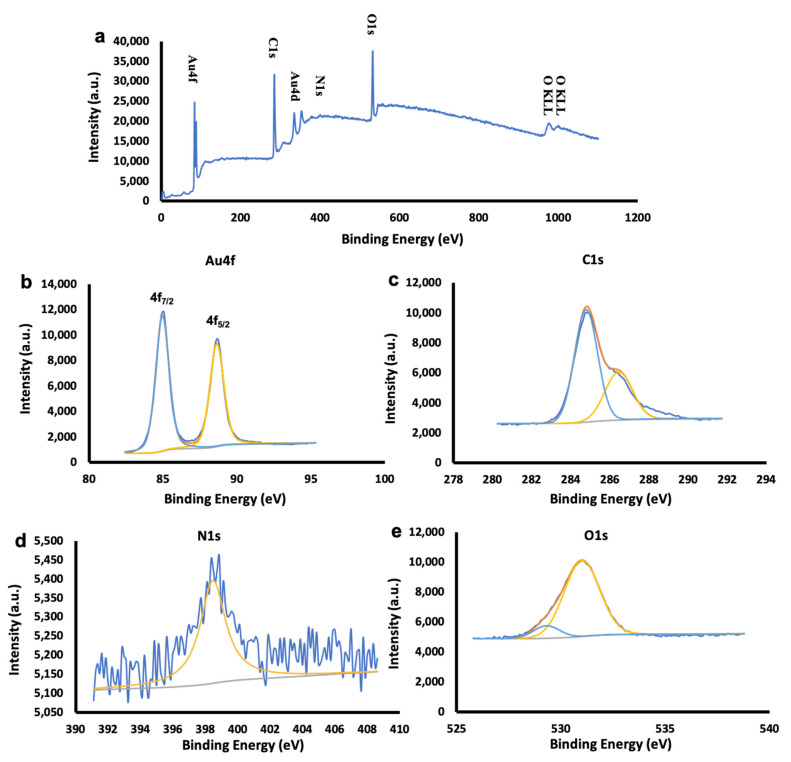
XPS analysis of Ch-AuNPIV (**a**–**e**). (**a**) Wide spectra, (**b**) Au spectra, (**c**) carbon spectra, (**d**) nitrogen spectra and (**e**) oxygen spectra.

Based on the survey observations (Figure 3 and Figures S2–S11), Table S1 indicates the Au 4f_7/2_,_5/2_ coupled peaks in the narrow binding energy spectrum along with Au 4d_5/2,3/2_ which are in close alignment with the values for metallic gold found in the literature [52]. In Table S2, which consists of aliphatic C-C with a non-oxygenated ring and hydroxyl carbon C-O representing the oxidation species, the peak positions of the deconvoluted C1 spectra are tabulated. It is known that the interaction between the carbon-bear radicals and decomposed OH groups increase the C-OH bonds, which can be seen in all the samples due to increasing synthesis temperatures [53]. The redox reaction and the organic-inorganic interaction on the metallic surface due to the adsorption of organic components from the Chaga extract could have contributed to the appearance of N1s peaks on all the samples. Incidentally, XPS spectra of the Chaga extract (Figure S2) reveal the presence of C1s, N1s, O1s, Si_2p/2s_ and K_2p_/K_2s_ in the survey spectra. It was possible to fit the deconvoluted carbon spectrum into three components, C-C at 284.8 eV, C-O at 286.5 eV and C=O at 287.9 eV. Neutral amino group -NH_2_ or N-H bonds can be attributed to the N1 peak at 398.8 eV. O=C-OH and OH can be applied to the peak intensities at the O1s core stage, 529.5 eV and 531.5 eV, respectively. The presence of C, K and N signals in the extract are confirmatory of the rich biomolecular composition of the Chaga extract, which would have contributed to the gold nanoparticle synthesis and capping. A capping by biomolecular components can play crucial roles in the future bio-applications of the materials, such as elevating the compatibility or rendering toxicity, which could be utilized against diseased cells, such as cancers.

In order to better explain its intended use, it is necessary to establish the chemical constituents of a natural extract. Chaga is rich in various biologically active biomolecules. A few were analyzed and their correlative presence in the Ch-AuNPs also investigated. The total protein, tannins and ascorbic acid contents were assessed (Table 2 and Table S3). Tannins are astringent, bitter plant polyphenolic compounds that bind to and precipitate proteins and various other organic compounds. In several plant species, tannin compounds are widely prevalent, playing a key role during self-defense against predation, and perhaps even as pesticides, and in controlling plant growth [54]. 

It is tannins that give Chaga tea a distinctive taste. It was determined that the tannin content was 1.5 mg/mL in the extract. Vitamin C (L-ascorbic acid) is a water-soluble vitamin which is an important dietary ingredient because humans, unlike most animals, are unable to synthesize it on their own. Epidemiological research indicates that higher intake of fruit and vegetables is associated with a lower incidence of most forms of cancer, possibly partly due to their high vitamin C content [55]. Vitamin C can restrict the formation of carcinogenic substances, such as nitrosamines, modulate immune response and potentially reduce oxidative damage that can lead to cancer through its antioxidant role [56,57,58]. In vitro and in vivo, high-dose ascorbate has cytotoxic effects on cancer cells, rendering it a pro-oxidative compound that catalyzes the synthesis of hydrogen peroxide in tissues rather than acting as a radical scavenger [58,59]. Ascorbic acid is commonly dispersed in plant cells and has been known as reducing and stabilizing agent in synthesis of gold nanoparticles [38]. It was found that the aqueous extract had 2.6 μg/mL of ascorbic acid. Additionally, the overall protein content of the extract was estimated to be 1.6 mg/mL, suggesting that the extract contained a significant amount of proteinaceous compounds. These biomolecules could have definitely played a crucial role in the AuNPs synthesis. The chemical constituent measurement shows that compounds, such as tannins, proteins, and ascorbic acid, are also present in the Ch-AuNPs (Table 2 and Table S3). The presence of such a diverse mixture of biomolecules is bound to render bioactivity to the usually inert AuNPs, which could be harnessed for desired bio-applications.

### 2.4. Cytocompatibility of Ch-Extract and Ch-AuNPs

**Figure 4:**
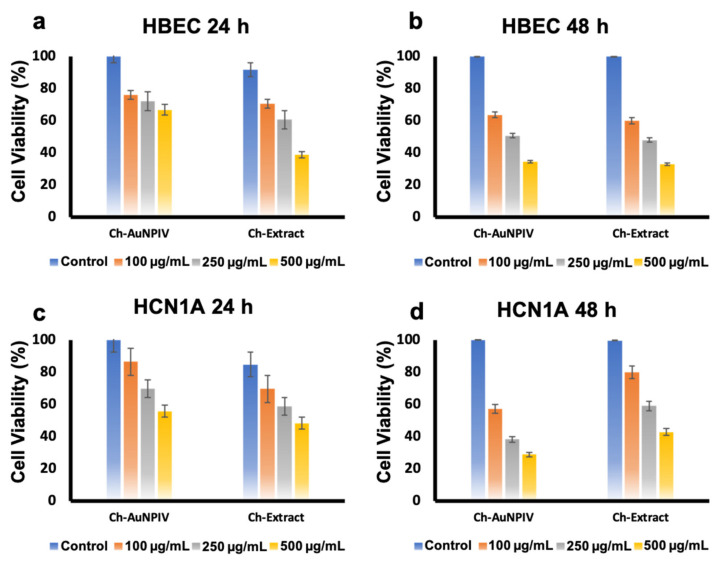
Cytocompatibility/toxicity analysis of the Ch-AuNPIV on normal brain cell lines. It could be observed that the NPs were clearly exerting toxicity to the cells (**a**,**c**), which was pronounced at 48 h observations (**b**,**d**).

It was found that both the Ch-AuNPIV and the extract caused significant toxicity in normal cells (HBEC and HCN-1A) (Figure 4a–d). At the highest concentration tested, the viability was 30 % for both nanoparticle and extract in the instance of HBEC (Figure 4b) after 48 h incubation. With 100 µg/mL, viability was found to be higher (60 %), indicating a dose-dependent impact. At 500 µg/mL concentration, the viability of HCN-1A after 48 h was determined to be 30 % and 40 % for Ch-AuNPIV and extract, respectively (Figure 4b). 

**Figure 5:**
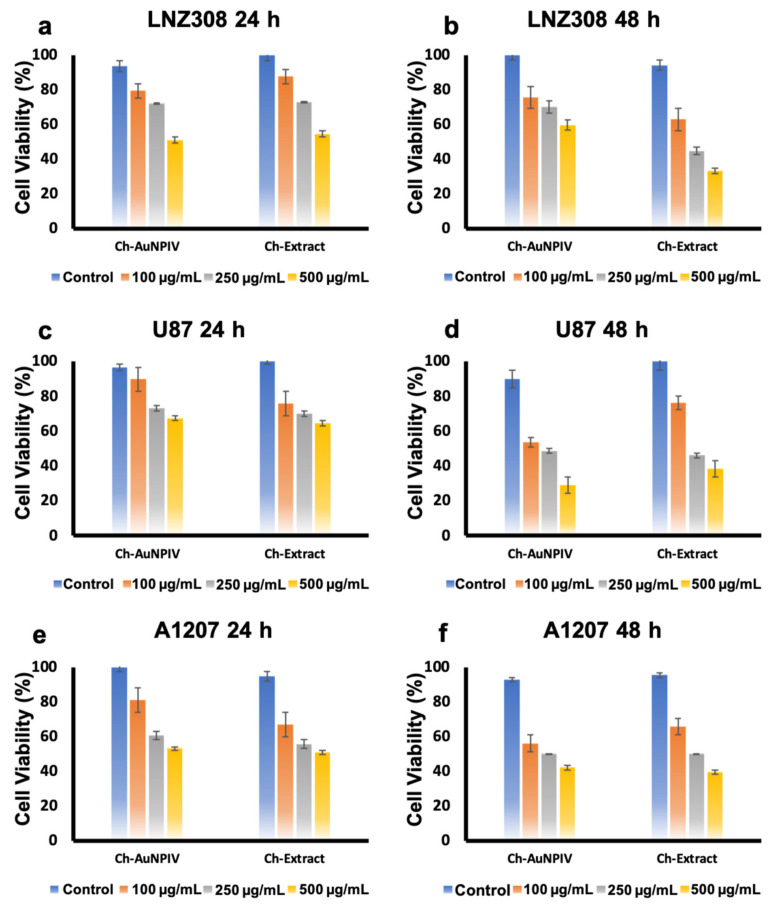
Cytocompatibility/toxicity analysis of the Ch-AuNPIV on brain cancer cell lines. Similar to that of normal cell lines, it could be observed that the NPs exert toxicity to the cells (**a**,**c**,**e**), which gets more pronounced with increase in incubation time (**b**,**d**,**f**).

With the brain cancer cell lines (Figure 5a–f), similar observations as that of normal cells was recorded. LNZ308 was the most resistant, with viability nearing 60 % even at 500 µg/mL of Ch-AuNPIV (Figure 5b), while U87 was the most susceptible (30 %) after 48 h (Figure 5d). A1207 also displayed a reduced viability on treatment of 500 µg/mL of Ch-AuNPIV (Figure 5f). The Ch-extract and Ch-AuNPIV were found to have a deleterious influence on the viability of practically all cell lines. The presence of biomolecules on their surface from the extract could explain the effect with Ch-AuNPIV. Furthermore, since the toxicity was observed equally in the normal cell lines as well, a regimen where specific moieties are utilized to target especially the cancer cells (targeted therapy) can be considered. It is a well-established fact that the biosynthesized nanoparticles have an inherent tendency of exerting bioactivity against various life forms including bacterial, viral and human [60]. Though the bioactivity largely depends on the concentration of NPs used, shape, size and surface charge, in the case of biosynthesized NPs, the reducing biological source also plays a major role in determining the toxic/safety profile of the material. These observations are in relation to the biomolecular coating on the respective nanomaterials’ surfaces. Interestingly, these observations have both positive and negative effects on the growth and proliferation of the cells under consideration. These effects depend on the biomolecules displayed on the surface of the nanomaterials which govern their interaction and final activity on the cells [48,61,62]. Our observations with Ch-AuNPs show a similar pattern, however the toxicity was evident in both normal and cancer cell lines. Though it is exciting to learn of the anti-cancer activity of the Ch-AuNPs, the relative toxicity to normal brain cells is of concern. This particular concern can be circumvented by providing a biocompatible coating to the Ch-AuNPs and by specifically delivering the NPs to the cancer cells utilizing a targeting scheme [30].

### 2.5. Photothermal Properties of Ch-AuNPs

**Figure 6:**
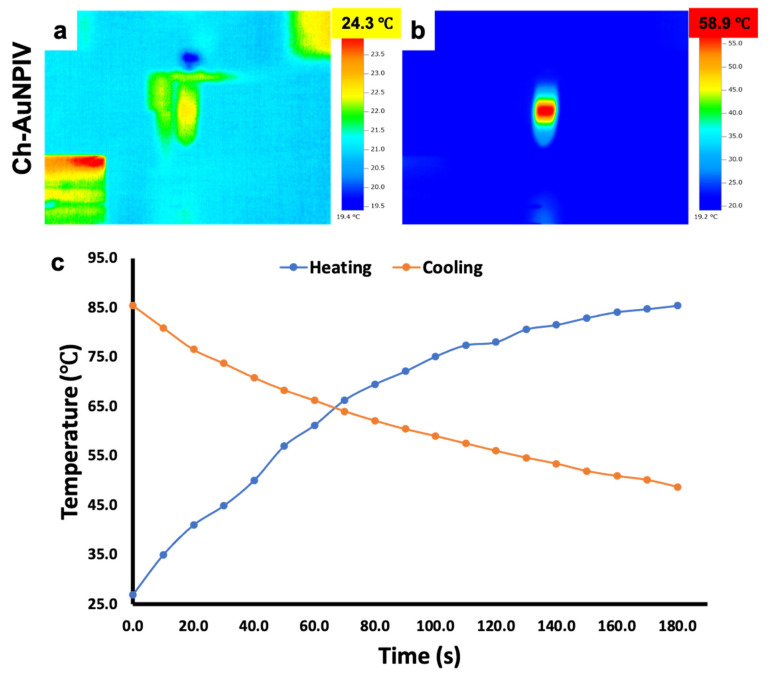
Photothermal responsiveness of the Ch-AuNPs under an external stimulus of NIR irradiation. (**a**) Depiction of the dispersion before the laser irradiation. The temperature rose rapidly once the NP solution was irradiated with the laser (**b**) which proves the known concept of AuNPs LSPR activity. (**c**) Furthermore, the heating and cooling studies revealed a rapid rise and gradual decrease of temperature, which could be beneficial under in vivo scenario where the temperature remains lethal in the case of a large solid tumor mass.

When Ch-AuNPs were irradiated with an NIR laser (Figure 6a,b, Figures S12 and S13), the conduction band electrons experienced synchronized oscillations, which cause the applied light to be absorbed or scattered. This phenomenon is transformed into heat energy, which is then dispersed into the surrounding media, in this case PBS, and detected by an infrared thermal camera. The temperature rise of the Ch-AuNPs in response to laser irradiation was in the range of 43–78 °C (Figure 6b, Figures S12 and S13). After 1 min of irradiation, all AuNPs (except V and IX) had a final temperature of more than 50 °C. The ultimate temperatures of Ch-AuNPV and Ch-AuNPIX were 44.2 °C (Figure S12e1) and 43.2 °C (Figure S13d1), respectively. It is worth noting that for PTT to be a successful anti-cancer therapy, a steady localized high temperature is sufficient to kill cancer cells. Consequently, even though Ch-AuNPV and Ch-AuNPIX had lower ultimate temperatures, they are still efficient PTT agents. To negate any impact from the solvent system, PBS alone was irradiated, but no thermal reaction was elicited, and the temperature remained unchanged (Figure S14).

Furthermore, the heating and cooling experiment indicated that the temperature rapidly increased during the time the laser was in switched on condition, followed by a consistent and gradual (not rapid) reduction after the laser was switched off (Figure 6c). It could be observed that even after 3 min (post laser switch off), the temperature remained around 45 °C. This leads us to infer that the surrounding media retains/keeps the temperature constantly high enough for a specific amount of time, which might be useful in the PTT of larger solid tumors, in theory.

Generally, gold nanoparticles are known to possess SPR optical property. This property helps in converting the incident photon energy (in our study, NIR light) into thermal energy [63]. We utilized NIR light due to its deeper penetration with less loss of light scattering in tissues (considering future in vivo approach). The observations seen in our study positively affirmed the conversion of NIR laser energy into heat by Ch-Au NPs. The quick rise of temperature is attributed to the fast plasmonic heat generation, whereas the cooling profile indicated slow dissipation of heat and follows the Newtonian cooling post laser off [64,65]. The variations in the measured temperatures between samples can be attributed to the variance in the composition of nanoparticle coating. Though Ch-AuNPV and Ch-AuNPIX exhibited maximum temperature rise post NIR exposure of 44.2 °C and 43.2 °C, respectively, it is well known that cancer cells are characterized by being sensitive to heat in comparison to normal cells, especially at temperatures higher than 42 °C. It is established that the temperature maintained at 42–45 °C for about half an hour can cause irreversible cellular damage and cell death. Therefore, the induced temperature increase seen in Ch-AuNPV and Ch-AuNPIX, with low photothermal conversion efficacy could also be potentially used to initiate irreversible damage to either cancer cells, tissues or both [66].

### 2.6. Anti-Cancer Photothermal Therapy by Ch-AuNPs

**Figure 7:**
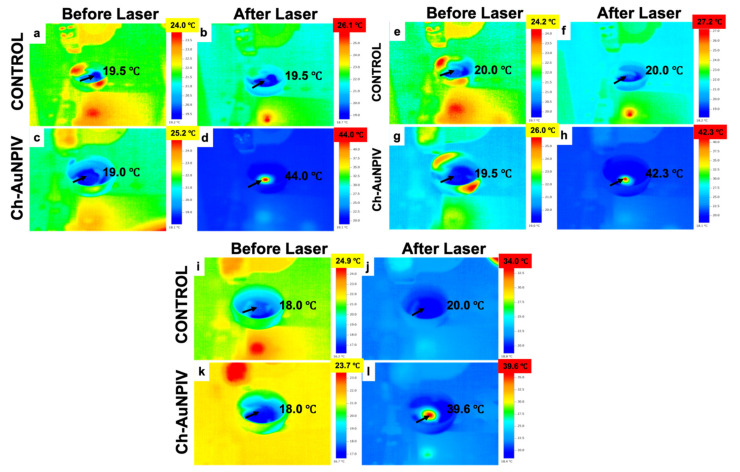
In vitro PTT of brain cancer cells. (**a**–**d**) A1207 cells, (**e**–**h**) LNZ308 cells, (**i**–**l**) U87 cells. It could be observed that the temperature of cells treated with Ch-AuNPs and further irradiated with an NIR laser, rose considerably enough to negatively affect the cell viability.

Once the photothermal properties of the Ch-AuNPs had been established, their PTT effects on cancer cells was tested (Figure 7a–l). The temperature in the Ch-AuNP-treated cells increased rapidly, as evidenced by infrared thermal imaging (Figure 7d,h,l). The temperature reached 44 °C in A1207 cells (Figure 7d) and 42.3 °C in LNZ308 cells (Figure 7h) after 1 min of NIR laser irradiation. In the case of U87 cells, however, the temperature was 39.6 °C (Figure 7l).

**Figure 8:**
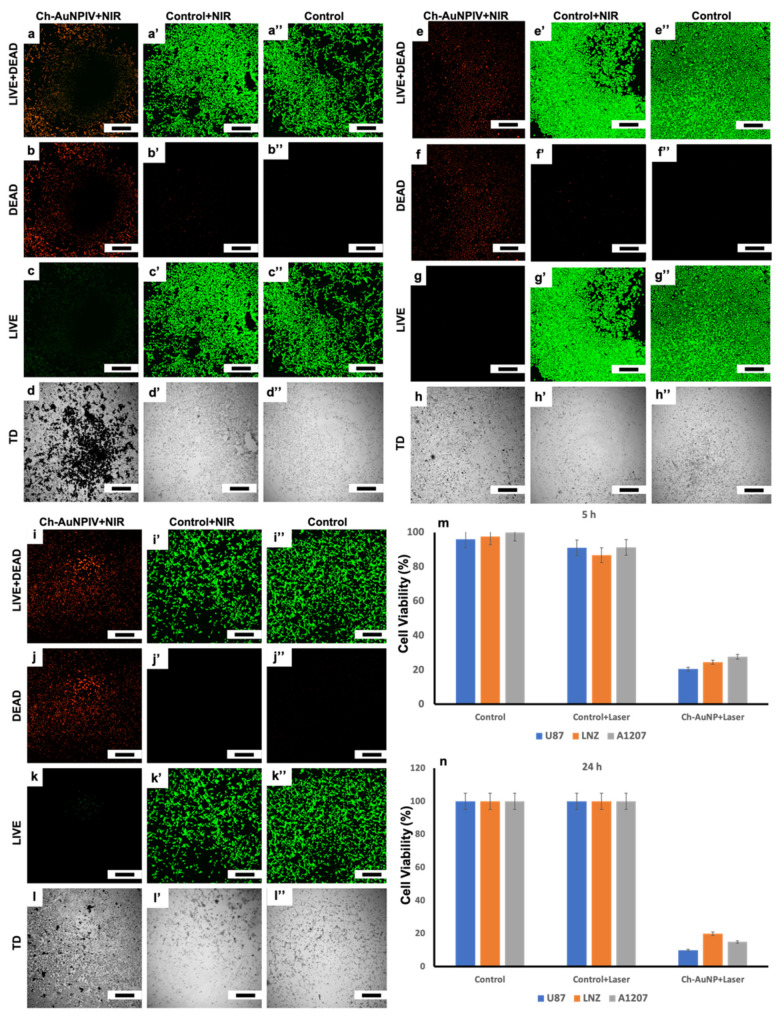
Post-NIR PTT observations revealed that most of the cells (Ch-AuNPs treated) were dead (**b**,**f**,**j**) with very few cells remaining metabolically active (**c**,**g**,**k**) Furthermore, cell viability assessment revealed that the cancer cells continued to die without any traces of relapse (**m**,**n**). (**a**–**a”**,**e**–**e”**,**i**–**i”**) Combined images of live (calcein) and dead (EthD-1) fluorescent cells. (**b**–**b”**,**f**–**f”**,**j**–**j”**) Fluorescent images of dead cells (EthD-1). (**c**–**c”**,**g**–**g”**,**k**–**k”**) fluorescent images of live cells (calcein). (**d**–**d”**,**h**–**h”**,**l**–**l”**) Bright field images (TD-transmitted image) (scale bar = 500 µm). (**m**) Cell viability assessment immediately after the laser exposure (5 h). (**n**) Cell viability assessment post 24 h of laser irradiation.

Nearly all of the cells exposed to Ch-AuNPIV + NIR laser showed apoptotic features and a prominent red fluorescence due to the ethidium homodimer (EthD-1) (Figure 8b,f,j), which enters cells with damaged membranes and undergoes a 40-fold enhancement of fluorescence upon binding to nucleic acids, resulting in a bright red fluorescence in dead cells as observed under a CLSM. Due to properly functioning intracellular esterase, which enzymatically transforms the essentially nonfluorescent cell-permeant calcein AM to the highly fluorescent calcein, the control and control + laser cells displayed brilliant green fluorescence. All three cancer cell lines had viable cell concentrations of less than 30 % after NIR laser irradiation, with U87 being the most susceptible (Figure 8m). The viability was drastically reduced by 24 h (Figure 8n), which reveals that the cells are unable to relapse after the PTT. The Ch-AuNPs were particularly good at converting NIR laser light to heat energy, which was subsequently used to kill the brain cancer cells. It has to be noted that the incubation time of the Ch-AuNPs with the cells was only 4 h which was followed by a one-minute laser irradiation, which was enough to kill more than 75 % of the cells. These findings indicate that Ch-AuNPs are effective PTT agents.

It is to be noted that lower temperatures of photothermal therapy are therapeutically ineffective, whereas higher temperatures may cause adverse side effects and an optimal temperature of 42–45 °C is quite enough to sensitize and kill cancer cells [67]. A sustained heat of 39–44 °C killed the cancer cells effectively in our study. This concluded the efficient sensitization of cancer cells to thermal energy. Another interesting observation is that the elevated temperature in the irradiated zone is seen dissipating to the surrounding cells that could be visualized as spatiotemporal temperature distribution with the help of IR camera. This is interesting because the maintained therapeutic temperature would sensitize cancer cells in the surrounding area but not the normal cells, as normal cells are not sensitive to the stated therapeutic temperature. Furthermore, it is generally believed that increase in temperature makes the cancer cells susceptible to chemotherapeutics [68]. PTT induces expression of heat shock proteins that in turn alters the cancer cell’s plasma membrane permeability [69,70]. This increased permeability could make cancer cells susceptible to the biomolecules attached to the AuNPs (from Chaga extract), leading to more pronounced and effectual cell death due to PTT + chemo combinatorial modality.

## 3. Materials and Methods

### 3.1. Aqueous Extract of I. obliquus

Dried Chaga mushroom was broken down and ground in a mortar and pestle and stored at 4 °C (Figure S15). Furthermore, 50 g of Chaga powder was extracted with 250 mL of Milli-Q water in a Waring blender in a cold room (4 °C) for 1 min at high speed followed by a 5 min rest. This cycle was repeated 5 times. The resultant thick solution was then filtered with 0.2 μm vacuum filter unit (TPP) to separate out the solid and liquid fractions. The filtration process was continued for 24 h due to the thick nature of the solution. The final extract was dark brown in color. This extract was stored at 4 °C until further use. The extraction scheme is represented in Figure S16.

### 3.2. Synthesis of Ch-AuNPs

The Ch-AuNPs synthesis involved the reaction of 5 mL Chaga extract with different ratios of 0.001 M aqueous solution of HAuCl_3_·3H_2_O (Sigma, Shanghai, China). The reactions were performed in a shaking incubator (BioShaker, Taitec, Sausal, Austria) at 150 rpm set at 30–70 °C (Table 3) for 24 h.

Two different concentration ratios of extract and gold chloride solution, 1:1 and 1:2, respectively, were selected for this purpose. The solutions, post reaction, were centrifuged at 15,000 rpm for 30 min at room temperature to collect the Ch-AuNPs. The Ch-AuNPs were further resuspended in Milli-Q water and vortexed for approximately 1 min and centrifuged again at 15,000 rpm for 10 min. This process was repeated 3 times to remove the excess unreacted components of both the extract and the HAuCl_3_·3H_2_O. The final washed Ch-AuNPs were dispersed in Milli-Q water and stored at room temperature for further use. The synthesis process is demonstrated in Figure S17.

### 3.3. Characterization of Ch-Extract

The ascorbic acid, protein and tannins concentration in the extract were confirmed to finally determine the role of these constituents in the AuNP formation process. The ascorbic acid content was determined with the ascorbic acid assay kit (Sigma), while the total proteins were determined by the Pierce BCA protein assay kit (ThermoFisher Scientific, Shanghai, China) and the total tannin content was assayed with the tannin microplate assay kit (MyBioSource, San Diego, CA, USA). XPS was performed using ULVAC-PHI Quantes instrument with the binding energy of all spectra being recorded from 0 to 1000 eV with pass energies of 69 eV, 140 eV and 280 eV. AlKα was used for X-ray generation, for the high resolution and wide scan spectra, respectively.

### 3.4. Characterization of Ch-AuNPs

As a preliminary observation, the formation of nanoparticles was confirmed with their respective UV-Visible spectra using a Beckman Coulter Du730 UV-Visible spectrophotometer with a resolution 1 nm. Particle morphology as size and shape were assessed under a transmission electron microscope (TEM), JEOL 2100, operated at an accelerating voltage of 200 kV. Dynamic light scattering analysis was conducted to determine the hydrodynamic diameter and surface charge of the Ch-AuNPs using a Nano ZS zetasizer (Malvern, Malvern, UK) at 25 °C. The elemental configuration was determined by XPS using an ULVAC-PHI Quantes instrument with the binding energy of all spectra being recorded from 0 to 1000 eV with pass energies of 69 eV, 140 eV and 280 eV. AlKα was used for X-ray generation, for the high resolution and wide scan spectra, respectively. The biomolecular (ascorbic acid, protein and tannins) presence on the AuNPs was also confirmed following the same procedure as that of the Chaga extract. Ch-AuNPIV was chosen as the representative candidate nanoparticle for bio-related studies due to its size (see Table 1) and overall uniformity in shape (Figure 2).

### 3.5. Photothermal Properties of Ch-AuNPs

A highly monochromatic, collimated beam of NIR range (800 nm) (Chameleon Ultra diode-Pumped Mode Locked-Sub 200 Femtosecond Laser (Coherent 80 MHz repetition rate)) with a power of 2.027 W cm^−2^ (Laser power meter: VEGA, OPHIR, Japan) was utilized. The temperature variations were measured and analyzed with an infra-red (IR) thermometer (Thermal imager test 881-2 (Testo AG, Germany)). Ch-AuNPs (I–X) were dispersed in 1 mL phosphate buffered saline (PBS, Sigma Aldrich) at a concentration of 1 mg/mL. To determine the photothermal responsiveness of Ch-AuNPs, they were irradiated using the NIR laser of wavelength and power mentioned above for 60 s. Further, a heating and cooling profile of Ch-AuNPIV (chosen for its uniform morphological characteristics) was recorded for 180 s. In this experiment, the laser was irradiated on the Ch-AuNPIV for 180 s and temperature recorded every 10 s, after which the laser was switched off and again the temperature was recorded every 10 s. Furthermore, 1 mL PBS was irradiated with the laser as a control experiment to negate any effect of the solvent in temperature rise/fall.

### 3.6. Cytocompatibility and Anti-Cancer Studies of Ch-Extract and Ch-AuNPs

For the cytotoxicity/compatibility analysis, 4 mammalian cell lines were chosen—human brain microvascular endothelial cells (HBEC, Cell systems), human cortical neurons (HCN-1A, ATCC) and brain cancer (glioblastoma) cells (U87, LNZ308, A1207). The brain cancer cells were kindly provided by Prof. Kazuhiko Mishima, Saitama Medical University. All cells (except HBEC) were maintained in T25 flasks using DMEM (Gibco) supplemented with 10 % fetal bovine serum (FBS) and 1 % antibiotics in an incubator at 37 °C with 5 % CO_2_. HBEC were maintained in classic culture medium (Cell Systems) in an incubator at 37 °C with 5 % CO_2_. Medium was replenished every 2 days and the cells were subcultured once 90 % confluency was reached. Post confluency, cells were trypsinized (Trypsin-EDTA, Gibco), pelleted and approximately 10,000 cells were added to each well in 96-well plates (TPP) and cultured for 24 h prior to nanoparticle and extract exposure. All the experiments were conducted in triplicate. The controls were maintained devoid of any treatment, whereas test groups were treated with 100 μg/mL, 250 μg/mL and 500 μg/mL of the respective nanoparticle and extract. The plates were incubated for 24 and 48 h, after which the spent media was aspirated and fresh media was supplemented. The cell viability was assayed with PrestoBlue HS reagent (ThermoFisher Scientific) followed by fluorescence intensity measurement of the final product with a microplate spectrofluorimeter (SpectraMax i3x Microplate Reader). The viable percentage of cells was calculated for each group and plotted against the concentration of the test compounds (Ch-extract, Ch-AuNPs).

### 3.7. Anti-Cancer Photothermal Property of Ch-AuNPs

The cell culture methods were as described in the previous section. For the PTT studies the brain cancer cell lines were utilized. The cells were treated with Ch-AuNPIV at a concentration of 250 μg/mL for 4 h at culture conditions (37 °C with 5 % CO_2_). Post incubation period, the cells were washed with PBS (thrice) and fresh media was supplemented. The cells were then irradiated with the NIR laser for 1 min. After the laser exposure, the cells were tested for cell viability using PrestoBlue and live-dead cell viability reagents (Invitrogen). The visual observation of live and dead cell populations was determined in a high-speed confocal laser scanning microscope (CLSM, Nikon A1+, Tokyo, Japan).

## 4. Conclusions

In many regions of the world, Chaga has been utilized in folk and traditional medicine. It is claimed to be effective in the treatment of a variety of illnesses, including cancer. Chaga tea is high in antioxidants, which is considered as one of the most potent antioxidants, and has been shown to increase immunity. We were able to synthesize AuNPs with sizes less than 20 nm with minimum diversity in their shapes utilizing this amazing natural resource, in the belief that the bioactive components of the extract would impart their properties to the so synthesized AuNPs. The cytotoxicity studies revealed that the NPs had a toxic effect to both normal and cancer cells. This could be circumvented by utilizing a cancer cell-specific targeting scheme in the future. Additionally, combined with the cytotoxicity observations, and the 24 h post-PTT observations it could be inferred that the cell death, though accelerated by the thermal stimulus, was aided by the bioactivity of Ch-AuNPs, resulting in a dual modal, chemo-PTT, anti-cancer strategy.

## Figures and Tables

**Table 1:** DLS analysis of Ch-AuNPs and Ch-extract.

Sample	Zeta-Potential (mV)	Z-Average (nm)	Polydispersity Index (PI)
Ch-AuNPI	−24.8	41.4	0.4
Ch-AuNPII	−25.7	40.5	0.3
Ch-AuNPIII	−24.8	41.4	0.2
Ch-AuNPIV	−27.1	24.1	0.1
Ch-AuNPV	−26.7	33.0	0.2
Ch-AuNPVI	−23.3	27.2	0.3
Ch-AuNPVII	−23.2	53.0	0.3
Ch-AuNPVIII	−21.4	22.7	0.4
Ch-AuNPIX	−20.5	32.3	0.4
Ch-AuNPX	−20.9	32.8	0.5
Extract	−40.1	--	--

**Table 2:** Concentration of tannins, ascorbic acid and protein in the aqueous extract of Chaga and Ch-AuNPIV.

Sample	Tannin (mg/mL)	Ascorbic Acid (µg/mL)	Protein (µg/mL)
Ch-extract	1.58	2.61	1623
Ch-AuNPIV	0.55	0.26	226

**Table 3:** Ch-AuNPs synthesis parameters.

Sample	Extract: Gold Chloride (Ratio)	Temperature (°C)
Ch-AuNPI	1:1	30
Ch-AuNPII	1:2	30
Ch-AuNPIII	1:1	40
Ch-AuNPIV	1:2	40
Ch-AuNPV	1:1	50
Ch-AuNPVI	1:2	50
Ch-AuNPVII	1:1	60
Ch-AuNPVIII	1:2	60
Ch-AuNPIX	1:1	70
Ch-AuNPX	1:2	70

## Data Availability

Not applicable.

## Supplementary Materials

The following supporting information can be downloaded at: www.mdpi.com/article/10.3390/ijms23042292/s1.

## Abbreviations

NPs—nanoparticles, AuNPs—gold nanoparticles, Ch-AuNPs—Chaga extract reduced gold nanoparticles, NIR—near infra-red, AIDS—acquired immunodeficiency syndrome, SOD—superoxide dismutase, PTT—photothermal therapy, UV/Vis—ultraviolet-visible, SPR—surface plasmon resonance, TEM—transmission electron microscope, DLS—dynamic light scattering, mV - millivolt, Z-average—zeta average, PI—polydispersity index, XPS—X-ray photoelectron spectroscopy, HBEC—human brain microvascular endothelial cells, HCN-1A—human cortical neurons-1A, PBS—phosphate buffered saline, EthD-1—ethidium homodimer-1, CLSM—confocal laser scanning microscope, BCA—bicinchoninic acid, ATCC—american type culture collection, DMEM—dulbecco’s modified Eagle’s medium, FBS—fetal bovine serum.
