# Supplementary material for: Biological Synthesis of Bioactive Gold Nanoparticles from Inonotus obliquus for Dual Chemo-Photothermal Effects against Human Brain Cancer Cells
Source: Int J Mol Sci. 2022 Feb 18;23(4):2292. doi: 10.3390/ijms23042292 (PMC8880898; doi:10.3390/ijms23042292)
Supplement: Supplementary file 1 [file ijms-23-02292-s001.zip › ijms-1559181-supplementary.pdf]

Biological Synthesis of Bioactive Gold Nanoparticles from *Inonotus Obliquus* for Dual Chemo-Photothermal Effects against Human Brain Cancer Cells

Ibrohimjon Skukurov[1], M. Sheikh Mohamed[1,2#], Toru Mizuki[1,2], Vivekanandan Palaninathan[1], Tomofumi Ukai[1,2], Tatsuro Hanajiri[1,2], Toru Maekawa[1,2]

[1]*Graduate School of Interdisciplinary New Science, Toyo University, Kawagoe, 350-8585, Japan*

[2]*Bio-Nano Electronics Research Centre, Toyo University, Kawagoe, 350-8585, Japan*

SUPPORTING INFORMATION

# Corresponding author

M. Sheikh Mohamed. Ph.D.

Ph: +81 49 239 1273

Fax: +81 49 231 5117

E-mail: sheikh@toyo.jp

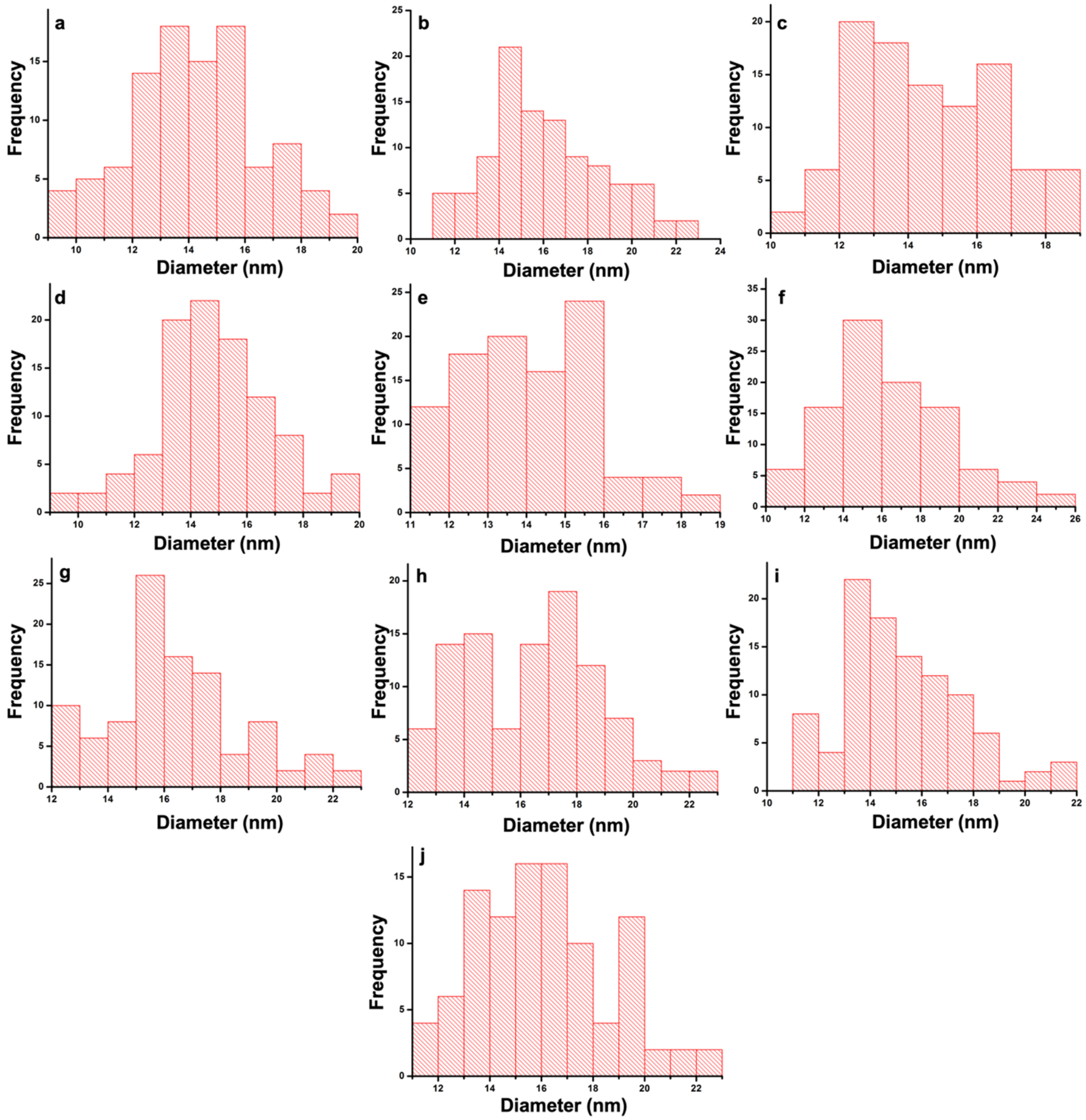

Figure S1. TEM micrograph-based size distribution of the Ch-AuNPs synthesized under various parameters. (a). Ch-AuNPI, (b). Ch-AuNPII, (c). Ch-AuNPIII, (d). Ch-AuNPIV, (e). Ch-AuNPV, (f). Ch-AuNPVI, (g). Ch-AuNPVII, (h). Ch-AuNPVIII, (i). Ch-AuNPIX, (j). Ch-AuNPX.

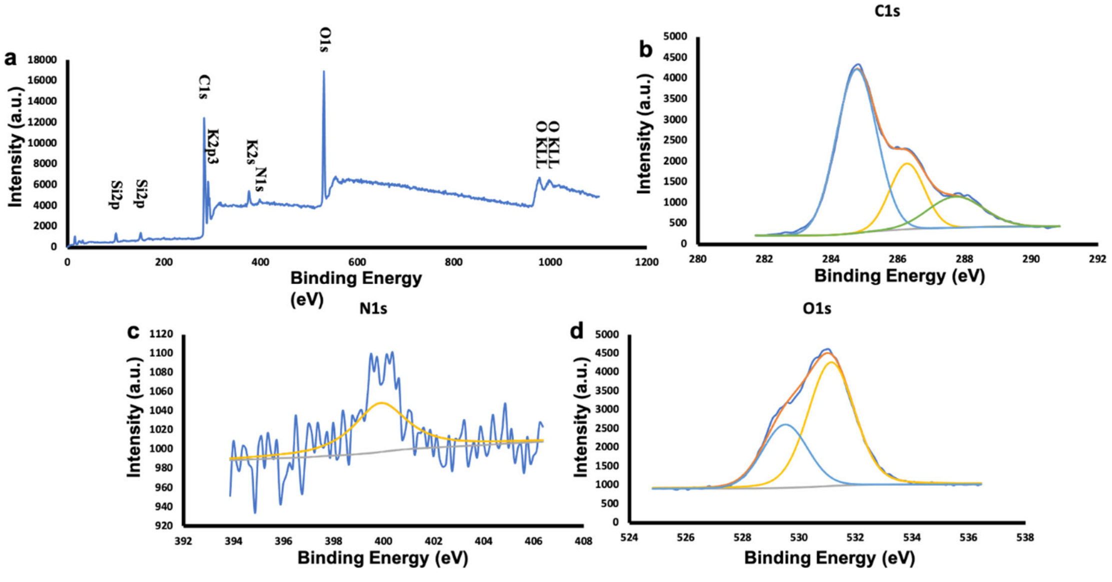

Figure S2. XPS analysis of Chaga extract. a. wide spectra, b. C spectra, c. N spectra, d. O spectra.

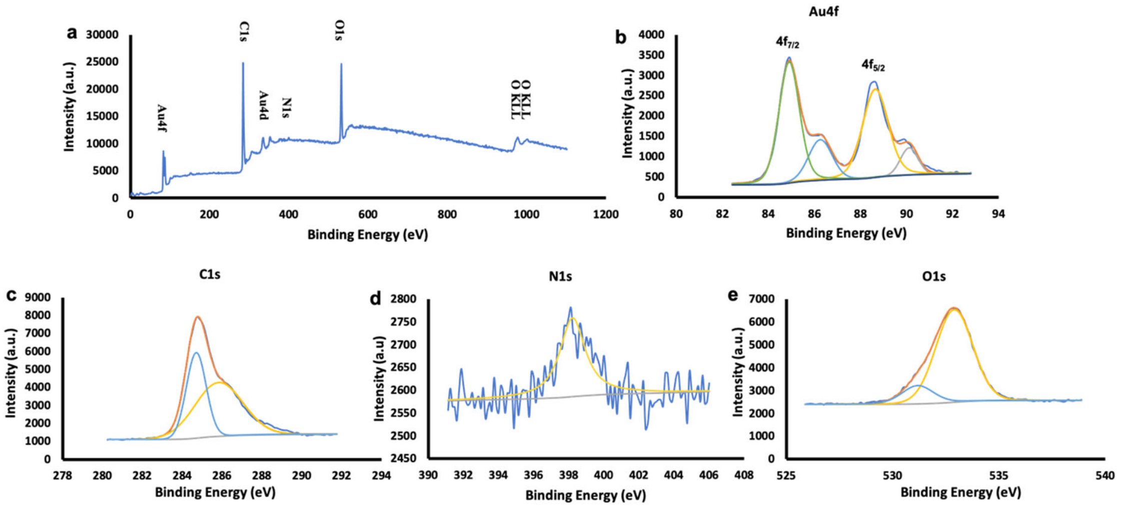

Figure S3. XPS analysis of Ch-AuNPI. a. wide spectra, b. Au spectra, c. C spectra, d. N spectra, e. O spectra.

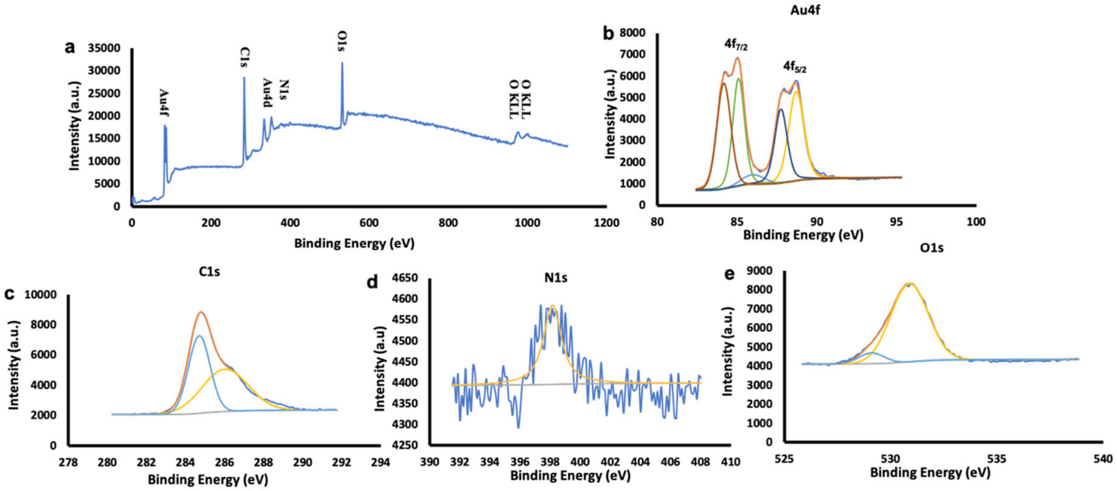

Figure S4. XPS analysis of Ch-AuNPII. a. wide spectra, b. Au spectra, c. C spectra, d. N spectra, e. O spectra.

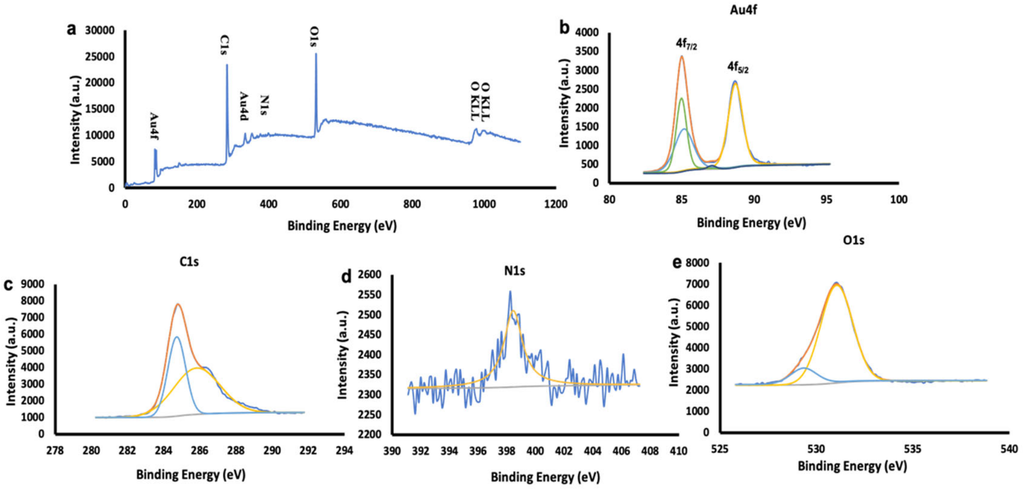

Figure S5. XPS analysis of Ch-AuNPIII. a. wide spectra, b. Au spectra, c. C spectra, d. N spectra, e. O spectra.

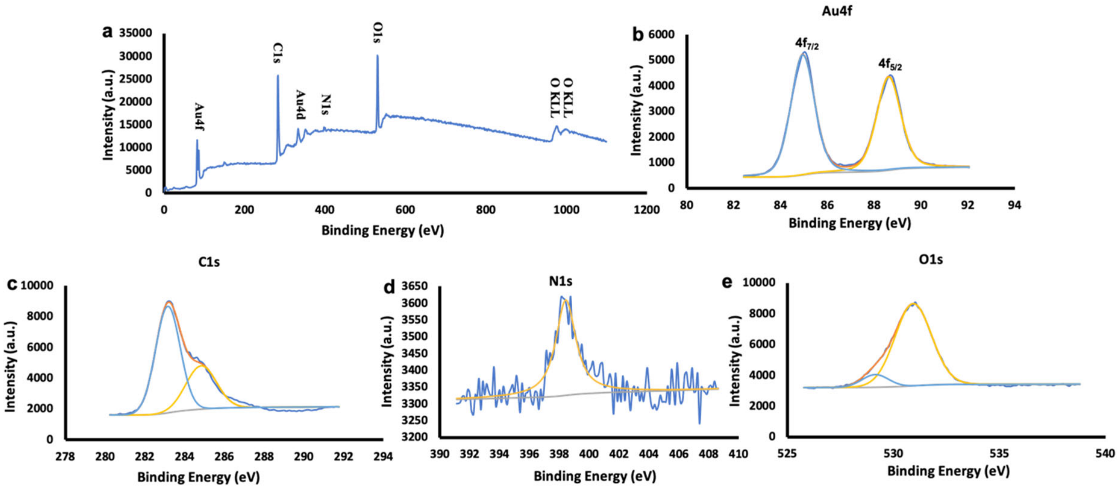

Figure S6. XPS analysis of Ch-AuNPV. a. wide spectra, b. Au spectra, c. C spectra, d. N spectra, e. O spectra.

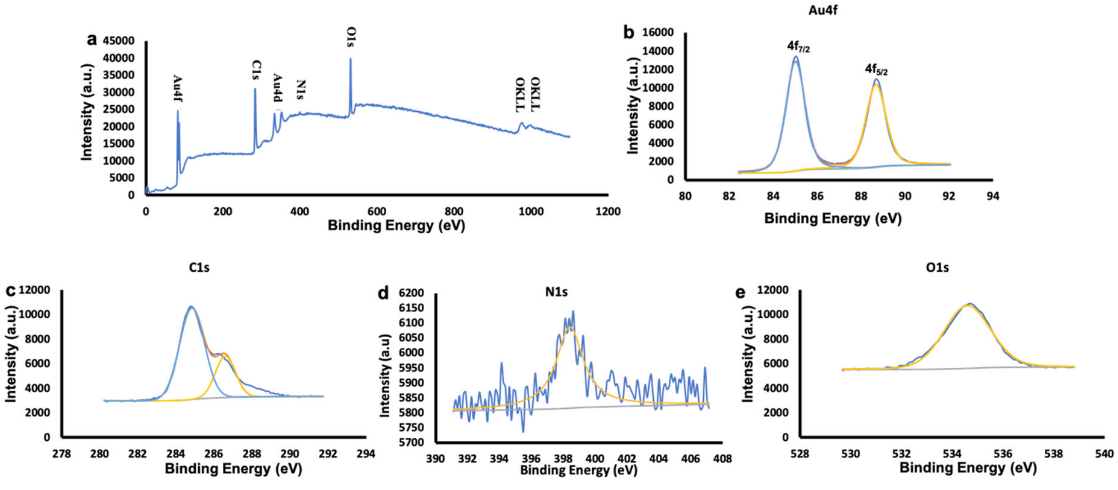

Figure S7. XPS analysis of Ch-AuNPVI. a. wide spectra, b. Au spectra, c. C spectra, d. N spectra, e. O spectra.

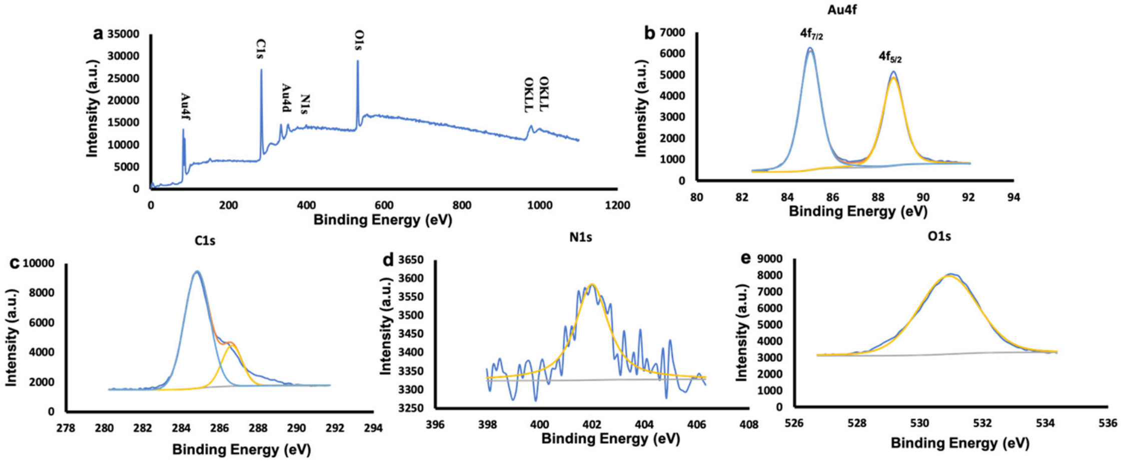

Figure S8. XPS analysis of Ch-AuNPVII. a. wide spectra, b. Au spectra, c. C spectra, d. N spectra, e. O spectra.

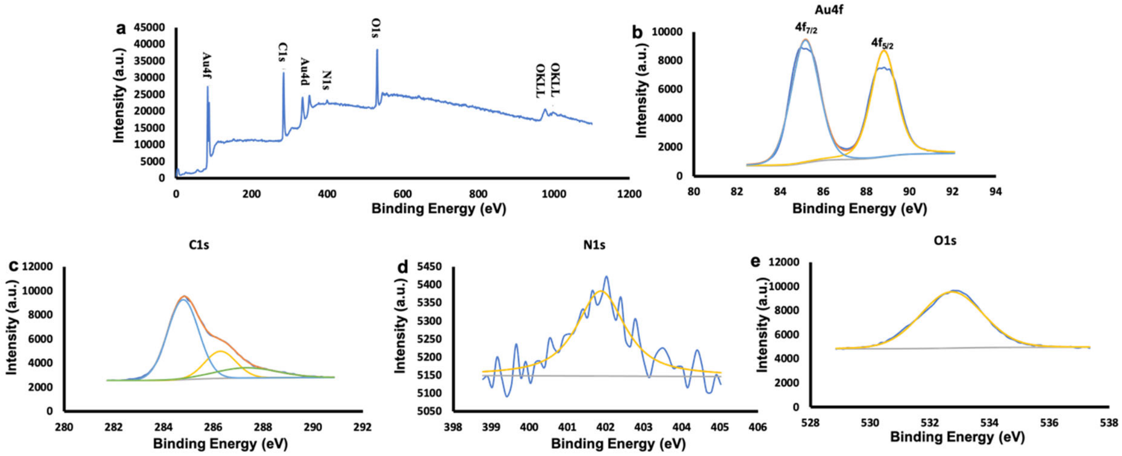

Figure S9. XPS analysis of Ch-AuNPVIII. a. wide spectra, b. Au spectra, c. C spectra, d. N spectra, e. O spectra.

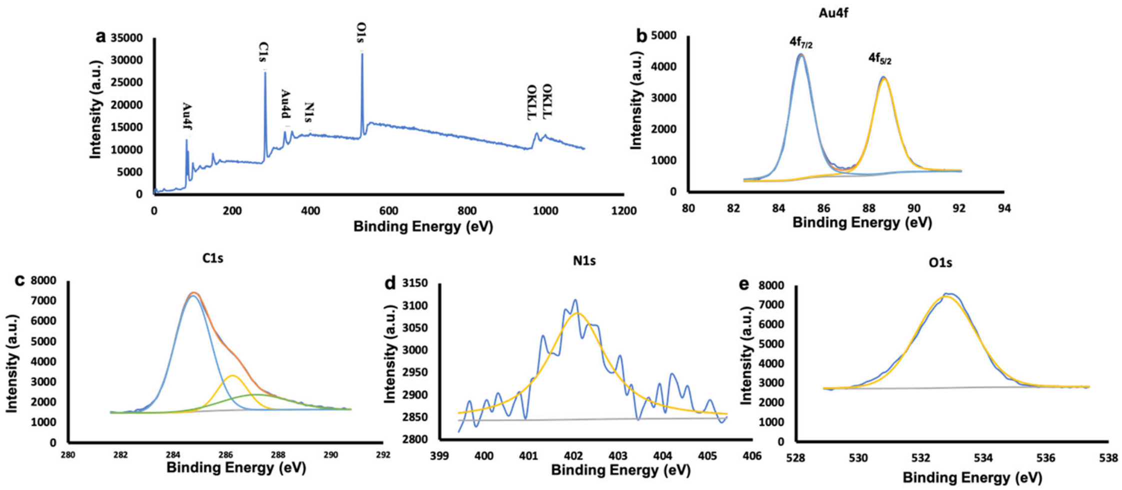

Figure S10. XPS analysis of Ch-AuNPIX. a. wide spectra, b. Au spectra, c. C spectra, d. N spectra, e. O spectra.

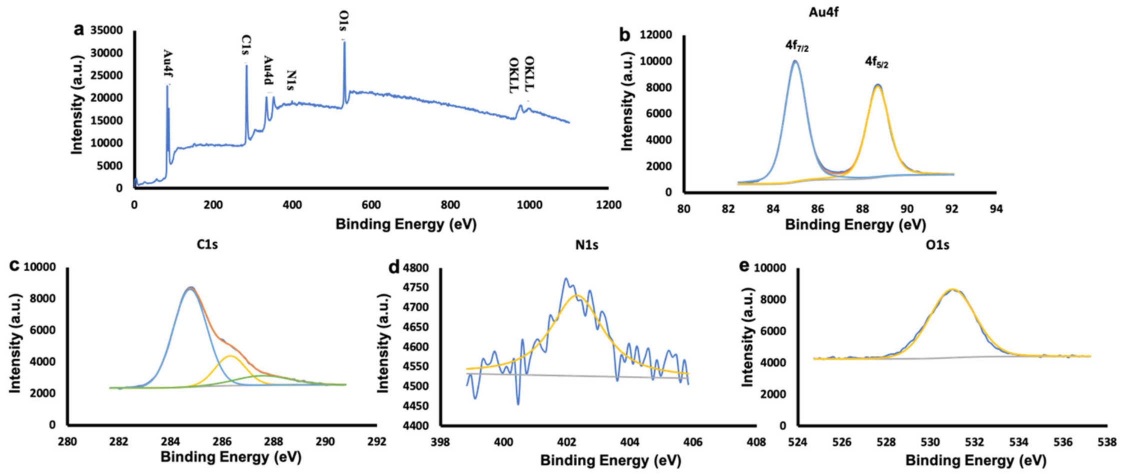

Figure S11. XPS analysis of Ch-AuNPX. a. wide spectra, b. Au spectra, c. C spectra, d. N spectra, e. O spectra.

Table S1. Au $4f_{7/2,5/2}$ coupled peaks in the narrow binding energy spectrum along with Au $4d_{5/2,3/2}$ of Ch-AuNPs.

Sample	$4f_{7/2}$	$4f_{5/2}$	$4d_{5/2}$	$4d_{3/2}$
Ch-AuNPI	84.4	87.4	334.4	352.4
Ch-AuNPII	83.0	86.0	334.0	352.0
Ch-AuNPIII	83.0	86.0	334.0	352.0
Ch-AuNPIV	84.0	88.0	335.0	353.0
Ch-AuNPV	83.0	86.5	334.0	352.0
Ch-AuNPVI	85.0	88.6	334.0	353.0
Ch-AuNPVII	84.5	88.6	335.0	353.0
Ch-AuNPVIII	83.0	87.0	335.0	353.0
Ch-AuNPIX	83.0	86.0	334.0	352.0
Ch-AuNPX	83.0	86.0	334.0	352.0

Table S2. Peak positions of the deconvoluted C1s spectra of Ch-AuNPs.

Sample	C-C	C-OR
Ch-AuNPI	284.8	286.1
Ch-AuNPII	284.8	286.2
Ch-AuNPIII	284.8	286.1
Ch-AuNPIV	284.8	286.3
Ch-AuNPV	283.8	285.0
Ch-AuNPVI	284.8	286.6
Ch-AuNPVII	284.8	286.5
Ch-AuNPVIII	284.8	286.4
Ch-AuNPIX	284.8	286.4
Ch-AuNPX	284.8	286.4

Table S3. Contents of tannins, ascorbic acid and protein in the Ch-extract and Ch-AuNPs.

Sample	Tannin (mg/mL)	Ascorbic acid (μg/mL)	Protein (μg/mL)
Ch-AuNPI	0.49	0.18	384
Ch-AuNPII	0.54	0.18	209
Ch-AuNPIII	0.48	0.19	628
Ch-AuNPIV	0.55	0.26	226
Ch-AuNPV	0.19	0.19	593
Ch-AuNPVI	0.53	0.15	435
Ch-AuNPVII	0.14	0.20	453
Ch-AuNPVIII	0.51	0.18	278
Ch-AuNPIX	0.38	0.23	697
Ch-AuNPX	0.11	0.15	453
Ch-extract	1.58	2.61	1623

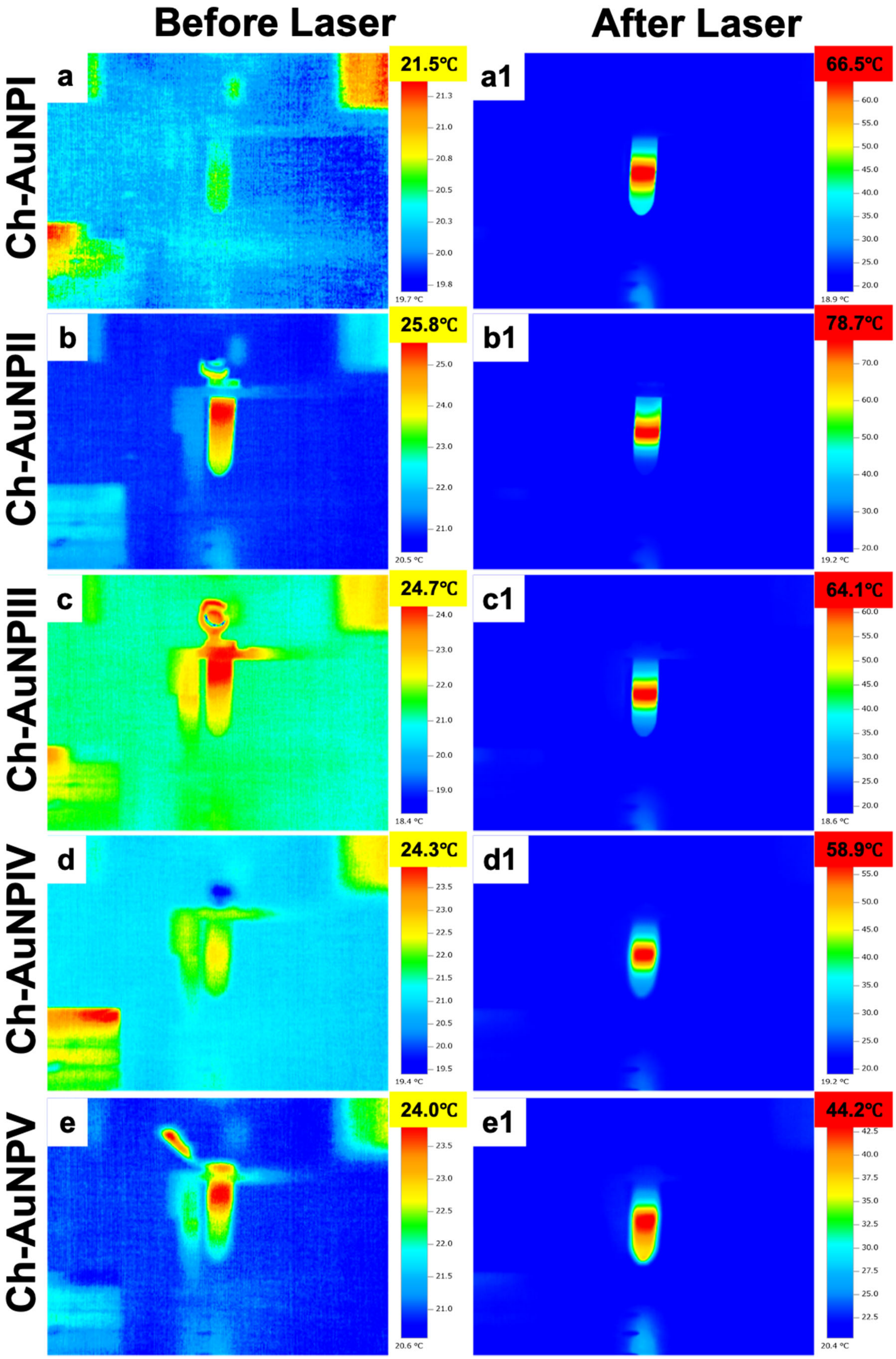

Figure S12. Photothermal responsiveness of the Ch-AuNPs (Ch-AuNPI-Ch-AuNPV) under an external stimulus of NIR irradiation.

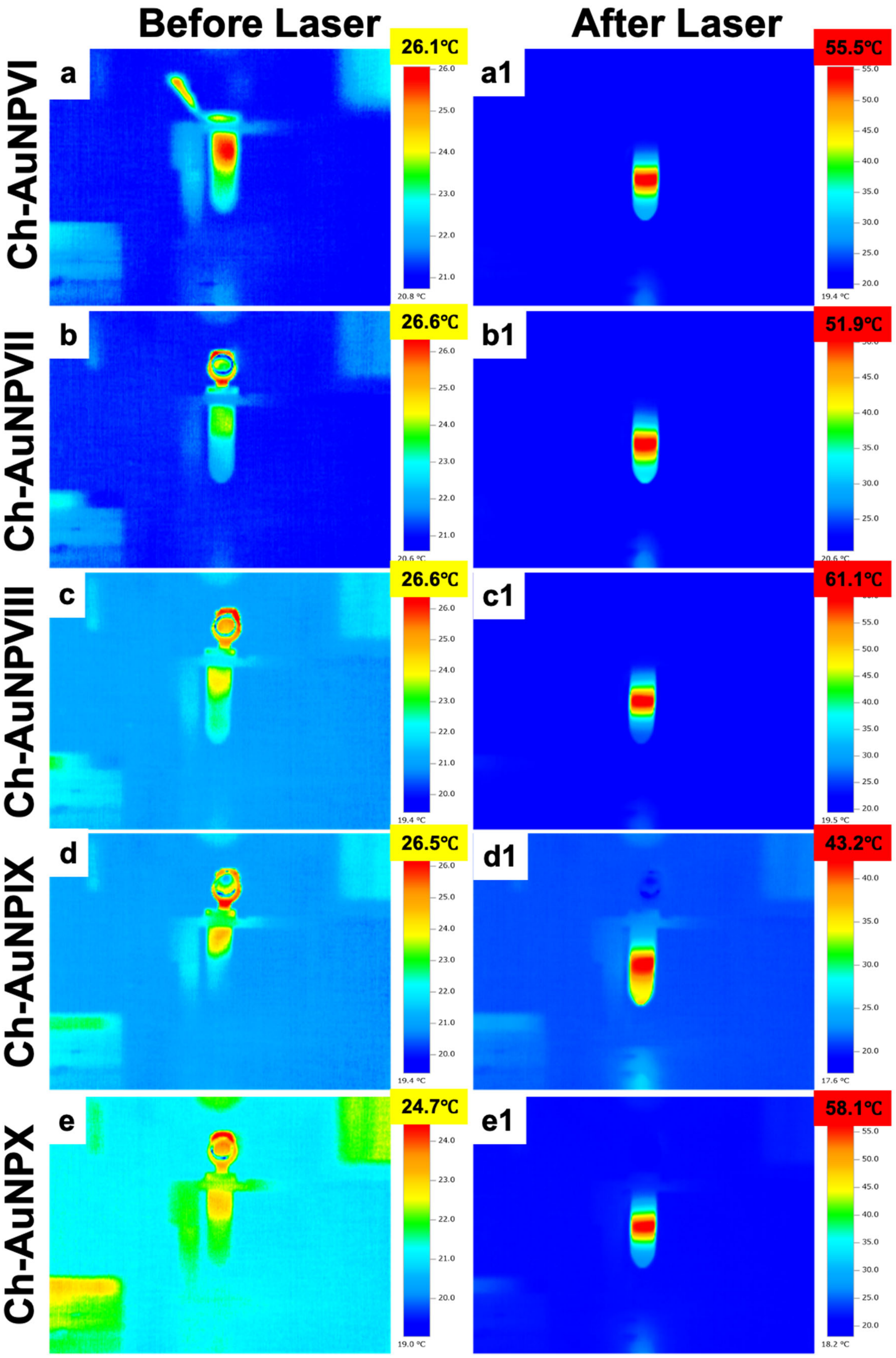

Figure S13. Photothermal responsiveness of the Ch-AuNPs (Ch-AuNPVI-Ch-AuNPX) under an external stimulus of NIR irradiation.

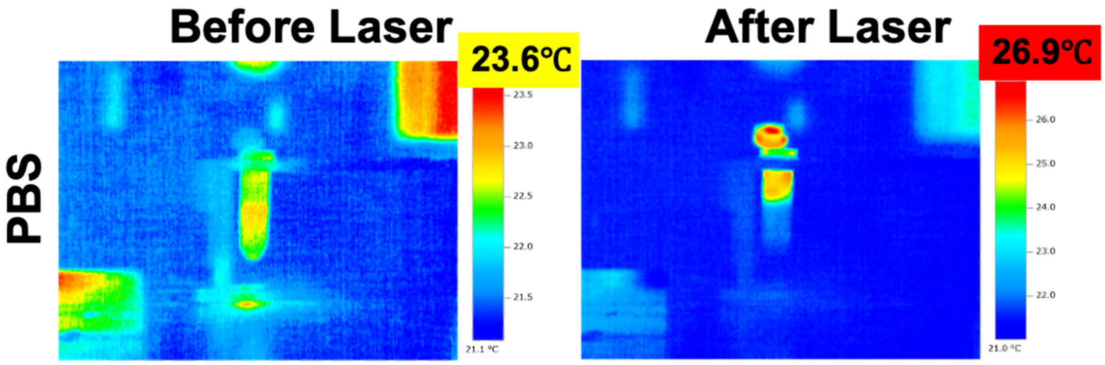

Figure S14. Photothermal responsiveness of PBS under an external stimulus of NIR irradiation.

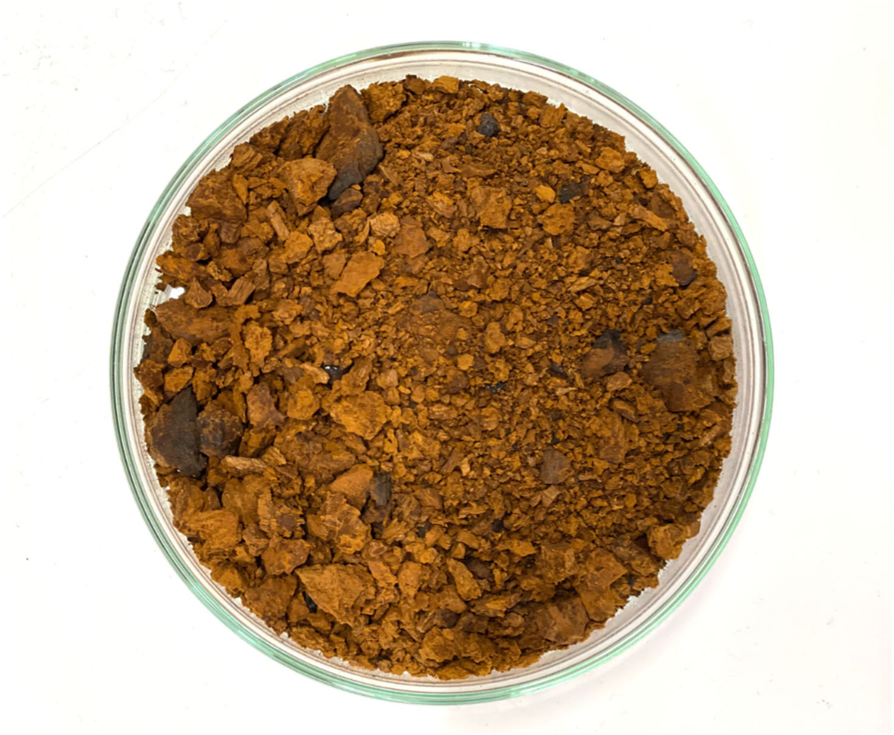

Figure S15. The broken up *I. obliquus* mushroom.

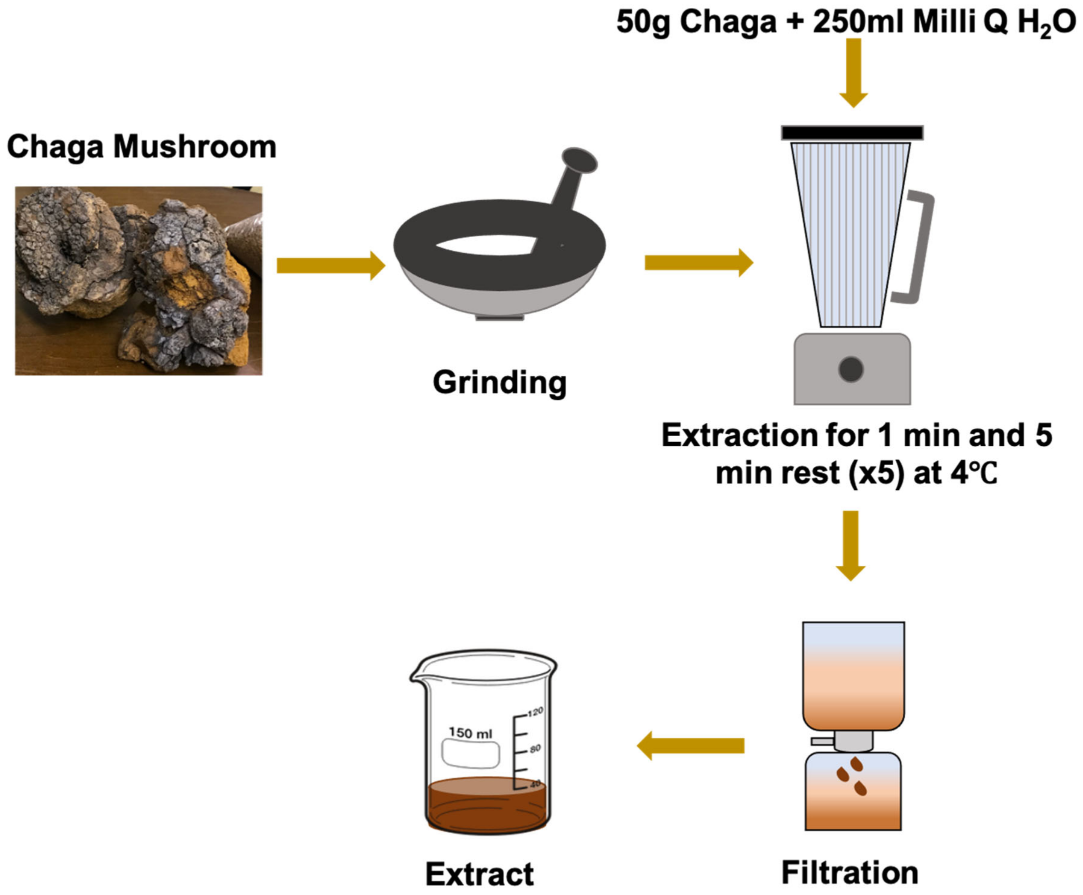

Figure S16. *I. obliquus* aqueous extraction scheme.

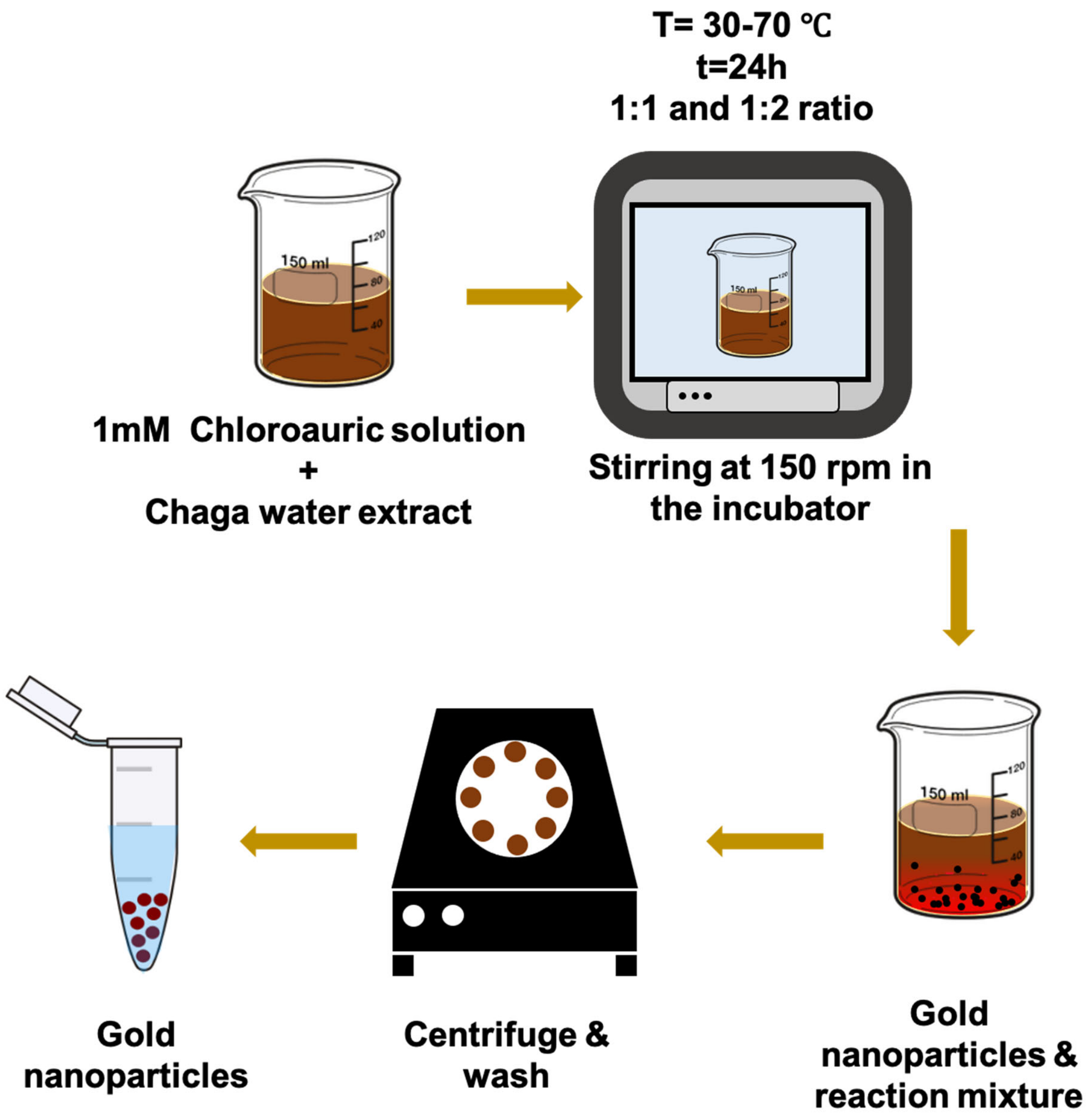

Figure S17. Scheme representing the synthesis of Ch-AuNPs from *I. obliquus* aqueous extract.
